# Retraction Note: Molecular mechanism and diagnostic marker investigation of endoplasmic reticulum stress on periodontitis

**DOI:** 10.1186/s12903-024-05158-w

**Published:** 2024-11-09

**Authors:** Qianqian Sun, Enqiang Zhu

**Affiliations:** Department of Stomatology, Sunshine Union Hospital, Yingqian Road, High-tech Zone, Weifang, 261000 Shandong China

**BMC Oral Health (2023) 23:135**

10.1186/s12903-023-02822-5

The Editor has retracted this article after concerns were raised about the data reported. The two datasets used in this analysis, GSE10334 and GSE16134, were each generated from the same archive of healthy and diseased gingival tissues, and as such there is a significant sampling overlap between the two. By using GSE10334 as a validation dataset for GSE16134, the authors do not appear to have accounted for these limitations. The Editor no longer has confidence in the reliability of the article’s results and conclusions.

The authors did not respond to correspondence from the Publisher regarding this retraction.

